# Utilizing Administrative Data to Focus Quality Improvement Efforts for Opioid Prescribing in an Integrated Health System

**DOI:** 10.5334/egems.203

**Published:** 2018-05-10

**Authors:** Priya Ramar, Daniel L. Roellinger, Jon O. Ebbert, Jenna K. Lovely, Lindsey M. Philpot

**Affiliations:** 1 Robert D. and Patricia E. Kern Mayo Clinic Center for the Science of Health Care Delivery, Mayo Clinic, Rochester, Minnesota, US; 2 Mayo Clinic College of Medicine, Rochester, Minnesota, US

**Keywords:** electronic health records, delivery of health care, quality improvement, health services research

## Abstract

This case study describes the use of multiple administrative data sources within a large, integrated health care delivery system to understand opioid prescribing patterns across practice settings. We describe the information needed to understand prescribing patterns and target interventions, the process for identifying relevant institutional data sources that could be linked to provide information on the settings for prescriptions, and the lessons learned in developing, testing, and implementing an algorithm to link the data sources in a useful manner.

## Context

Since the mid-1990s, prescription drug misuse has emerged as a significant threat to public health in the United States. Prescription opioid use is associated with the development of both opioid dependence and opioid use disorder [1,2,3]. More than 183,000 people in the U.S. died from overdoses related to prescription opioids between 1999 and 2015 [4]. Although the prevalence of opioid diversion (i.e., drug use for non-medically-indicated purposes or by individuals not prescribed the medication) is difficult to estimate [5], opioid overprescribing has been reported to occur, especially in the surgical practice [6,7]. Opioid overprescribing may increase the likelihood of drug diversion [8].

Limiting unnecessarily prescribed medications while improving pain management has been proposed as a key strategy for improving current opioid prescription practices [9]. In March 2016, the Centers for Disease Control and Prevention (CDC) released a guideline targeted at primary care clinicians prescribing opioids for chronic pain outside of active cancer treatment, palliative care, and end-of-life care [10]. Chronic use often begins with acute treatment of pain, and one of the guideline recommendations is to prescribe the “lowest effective dose” and “prescribe no greater quantity than needed.”

Guideline implementation may potentially reduce opioid over-prescribing and reduce opioid diversion, particularly where targeted implementation approaches are performed. In order to measure the impact of guideline implementation in clinical practice, reliable approaches for assessing baseline clinical provider prescribing practices are needed.

Our goal was to evaluate the distribution of opioids prescriptions across health care providers and settings in a large academic institution utilizing source data from an incorporated prescribing system which lacks accurate attribution of prescriptions to departments and providers. We created a deterministic match of each opioid prescription with a service provided in order to find the clinical setting, department, and provider.

## Case Description

This case study describes the use of multiple administrative data sources within a large, integrated health care delivery system to understand opioid prescribing patterns across practice settings. We describe the information needed to understand prescribing patterns and target interventions, the process for identifying relevant institutional data sources that could be linked to provide information on the settings for prescriptions, and the lessons learned in developing, testing, and implementing an algorithm to link the data sources in a useful manner. We describe challenges faced throughout this process, solutions employed, and resulting outcomes. These are also summarized in Table 1.

**Table 1:** Challenges, solutions, and outcomes presented through linking Decision Support System Administrative data with Enterprise Orders Prescribing data.

Challenge	Solution

Complexity of data sources	Review data and sources from both front-end user interface and also extraction in order to understand how nuances of each data source contribute to feasibility and impact on linkage Work with subject matter experts (clinical, pharmacy, pharmacy IT) to ensure appropriate use and interpretation of data
Opioid prescriptions that do not link to services with provider ID match	Develop multi-step algorithm to classify matches based on other variables; consult with informatics pharmacist and clinician through logistics of algorithm Classify patients by those who had all inpatient services, all outpatient services, both inpatient and outpatient services
Multiple services linked to opioid prescriptions using 3 day window for dates, including patients with opioids prescriptions linked to both inpatient and outpatient services	Remove non-prescribing departments (i.e., radiology, labs) and align data with institutional department groupings (i.e., primary care) to narrow scope of linking; consult with informatics pharmacist and clinician Assess and apply logic for chronology of dates for opioid orders and linked services
Data validation	Engage clinician and pharmacist for random case review and review of high dose orders

## Setting

Mayo Clinic is a large, integrated health care delivery system that serves more than 350,000 local, national, and international patients each year. Mayo Clinic has been using electronic resources for billing and patient care for many years. Mayo Clinic’s Decision Support System (DSS) aggregates coded clinical and financial data from various sources, and stores the information in a data warehouse, allowing for analyses to support clinical and financial decision-making. This administrative billing data contains information about patient hospitalizations and clinic visits, including timeframe, diagnoses, procedures, locations, and providers. Enterprise Orders Prescribing (EOP) is a tool that supports documentation and reconciliation of medications. EOP provides information on the ordering and signing providers, historical medications, prescription order, start and end dates, as well as information on the drug type, form of administration, amount dispensed, and medication instructions.

## Cohort

Ten opioids were of interest for this work: buprenorphine, codeine, fentanyl, hydromorphone, hydrocodone, methadone, morphine, oxycodone, oxymorphone, and tramadol. RxNorm [11] is a drug terminology system that provides a standardized nomenclature for clinical drugs and drug delivery devices. Since opioids can either be the primary substance within a medication or a supplemental ingredient (e.g., cough syrup with codeine), RxNorm contains the names of prescription and many non-prescription formulations that exist in the United States, including the devices that administer the medications. We used RxNorm to pull all relevant prescription orders with one of the 10 opioids listed as an ingredient from EOP.

Total morphine milligram equivalents (MMEs) were calculated using provided EOP data elements, and an Equianalgesic Dosing Ratio conversion table to standardize dose across drug types [12]. Team members worked with pharmacists to gain consensus on the conversion tool that was applied, and to calculate conversions for other formulations not available with the tool.

## Methods

### Data Approach and Algorithm

Due to the nature of the prescribing data, several exclusions were employed. “Historic” medications were excluded, as this notation is used for medications that the patient is no longer actively taking or those that were not issued as a result of the medication reconciliation process. Data exploration found that a number of opioid prescription orders were for the same drug, with the same order date, and for the same patient. Further examination of these situations indicated that the begin date and end date for these prescription orders were the same, and they were likely typographical entry errors that were immediately corrected. In these cases, we retained the most recent order for analysis.

In order to most effectively link opioid prescription orders to a prescribing provider and associated clinical department, prescribing data from EOP were linked to patient services in DSS using patient clinic number and prescription order date matched to DSS service lines within 3 days of the prescription order date. A range of 3 days was used in order to allow for capture of services billed on Monday that were conducted the previous Friday.

Using administrative location codes, prescription orders were grouped into Inpatient (IP), Outpatient (OP), and Emergency Department (ED) practice settings. Patients who were admitted to an IP service through the ED were included in the IP group. We developed an algorithm to match opioid prescription orders with services by separating patients into three categories: patients with all orders linked to IP services; patients with all orders linked to OP services; and patients with orders linked to both IP and OP services. Linking services within a range of three days of the prescription order date allowed multiple lines of services to be linked for most opioid prescription orders. We did an initial assessment of linked departments to remove clinical services that would not be prescribing opioids such as laboratory/pathology and radiology.

**Figure 1:**
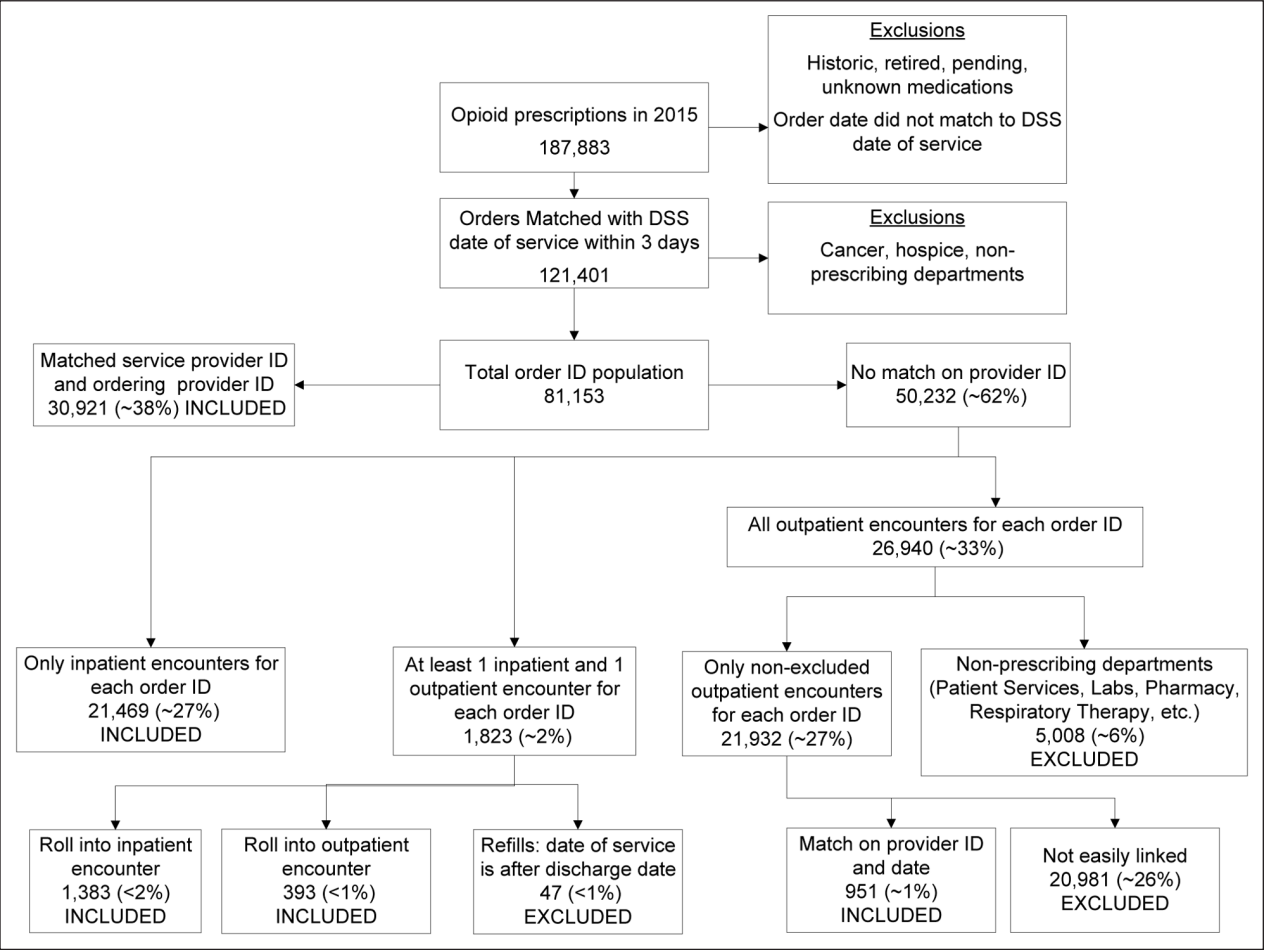
Linking Decision Support System Administrative data with Enterprise Orders Prescribing data (Footnote – the end of each line in the flowchart, starting with the total order ID population of 81,153, is marked as to whether that group was included or excluded).

Opioid prescription orders linked to only IP services were sorted by date and matched to the discharge service from the first chronological encounter. Those linked to only OP services were matched based on provider number from EOP and DSS. For prescription orders linked to both IP and OP services, if the OP service date was on or before the IP admit date, then we linked the order to the OP service; otherwise we linked the order to the IP service. Where the IP service was linked, if the prescription order date was after the discharge date, then that prescription order was considered a refill and subsequently excluded. Opioid prescription orders linked with the ED were classified separately. The process of linking EOP and DSS is shown in Figure 1.

### Assignment Validation

We tested the algorithm to validate assignment of practice setting, department, and provider with randomized manual chart reviews. Different validation approaches were used to ensure a comprehensive understanding of the data both from the prescription (EOP) side of the data, as well as the service and location (DSS) side of the data. Out of 81,153 EOP prescription orders for opioids in 2015, we linked 55,164 (68 percent) orders to a service line item in DSS and therefore to a patient visit or hospital discharge. Several groups of randomly selected test cases were researched with chart review by a physician on the team (JOE) to determine the accuracy of our IP and OP designations. A lead informatics pharmacist on the team (JKL) also conducted a review of randomly selected cases to help identify and remove abnormal MME values for specific orders.

We conducted an initial linkage validation for each clinical practice area (ED, IP, OP) separately, and aimed for a 10 percent threshold for appropriate matching before further investigation for linking error. We decided to target this validation to opioid prescription orders of more than 100 MME as those higher dosages would be of greater interest for targeting interventions. For orders originating from the IP setting, we randomly selected 12 records with greater than 100 MME for chart review. Chart review discovered that one out of 12 that was a questionable link, suggesting an 8 percent margin of error. A similar review for the OP setting found that four out of 12 (33 percent) were improperly linked. Further investigation into these links revealed that they were orders linked to both IP and OP services. In two cases, the OP setting was adjudicated to be more appropriate. The other two cases were unclassifiable as IP or OP. These cases were removed from our analytic dataset. We also discovered that many opioid prescription orders were proxied by a nurse or linked to a ‘Nursing Department.’ We adjusted our algorithm to pull the location codes for these prescriptions in order to link them to a department. An additional round of reviews for OP opioid prescription orders found missing links for those that were from Family Medicine, Primary Care Internal Medicine, and Pediatrics departments. At our institution, these departments are considered parts of the same Primary Care practice setting and we grouped them together as Primary Care. This resolved other similar questionable linkages for these departments. Our validation removed 2,980 (5 percent) of the orders.

### Visualization

**Figure 2:**
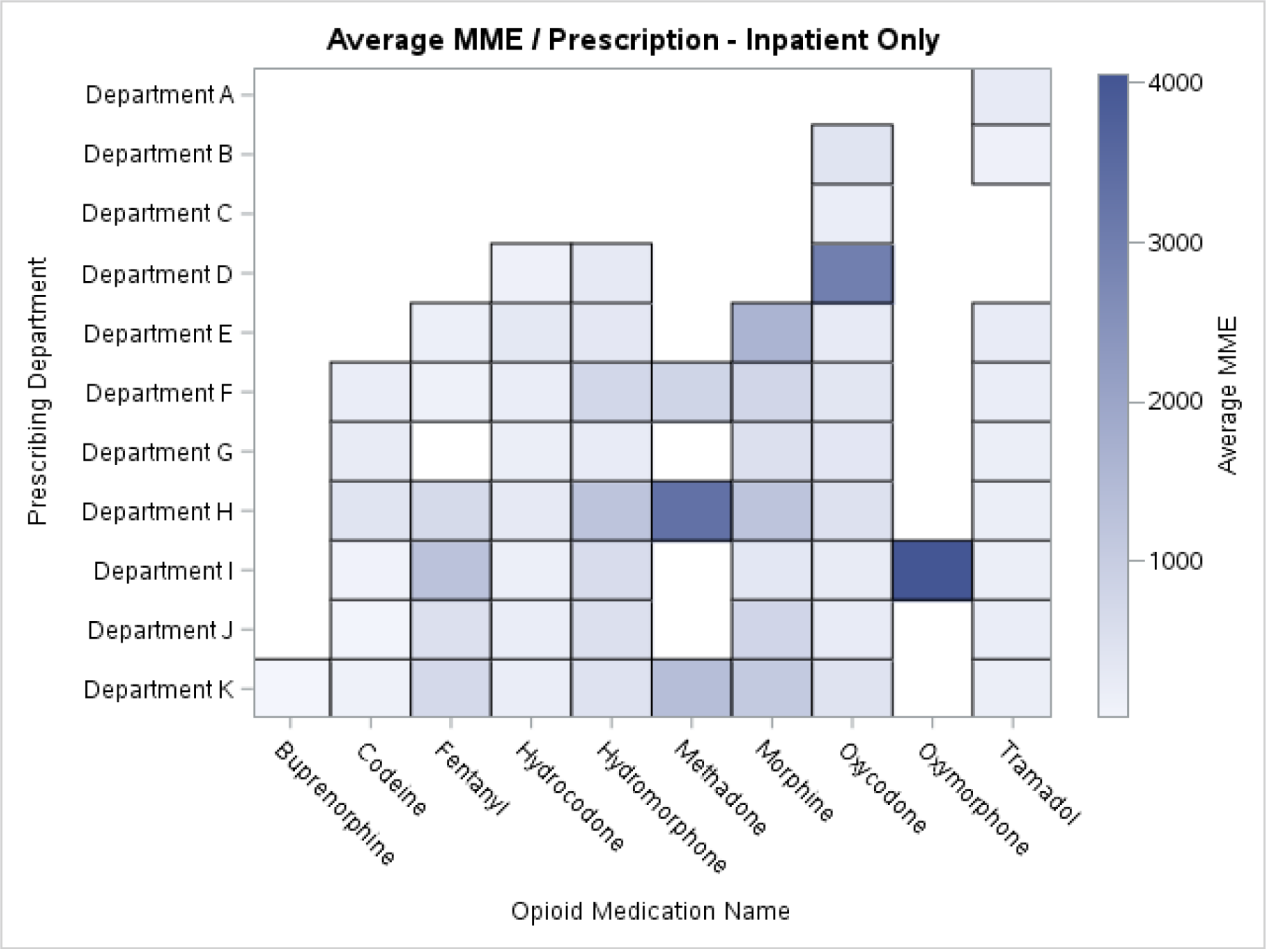
Heat Map of Opioid Prescribing Patterns in the Inpatient Setting.

**Figure 3:**
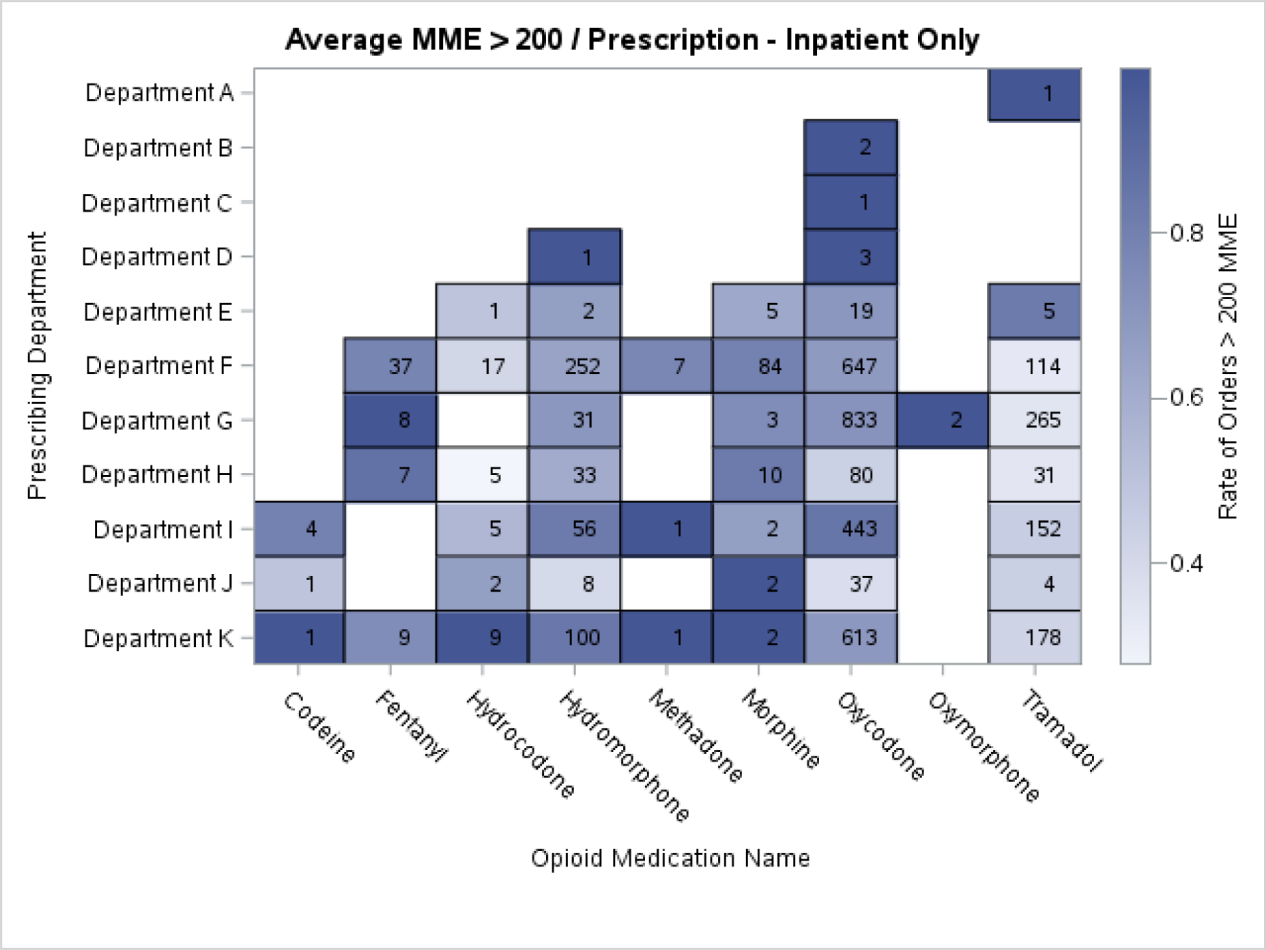
Heat map example of opioid prescribing patterns within the inpatient setting (orders with average Morphine Equivalent > 200).

The institution was interested in visual representations of opioid prescribing across the IP, OP and ED settings. We visualized our data as heat maps showing the level of opioid prescribing per department. Each linked prescribing department was shown along the vertical axis and each opioid medication was shown along the horizontal axis (Figure 2). The level of shading in each box represented the average MME per order by department or opioid, with darker shading representing higher average prescribed doses. Blank spaces indicated that no orders for that particular opioid were linked for the corresponding department. We constructed additional heat maps focusing on only those departments where the average MME per order was greater than 200, and indicated the number of prescription orders for each opioid in each department that comprised that average in order to identify departments with a greater volume of higher MME orders (Figure 3). A different approach was necessary for the emergency department since a single department heat map would not clearly present a sufficient amount of detail. We chose to present this data as a stacked bar graph displaying the ratio of opioid prescription orders with greater than 200 MME to those with less than 200 MME.

Discrepancy in the ability to assign individual opioid prescriptions to patient visits/hospital discharges was within our threshold of understanding due to the high-volume of patient interactions provided via non-visit care (e.g., online patient portal) employed within the care delivery model of Mayo Clinic. We classified orders based on practice setting: 50 percent of the orders were IP; 44 percent of the orders were OP; and 6 percent of the orders were ED.

### Targeting Intervention

Heat maps of opioid prescribing practices were shared with institutional leadership to target areas for improvement. Summary reports included overall prescribing patterns by department, then subset to patients with MME greater than those recommended by the state of Minnesota. These summary reports and analytic dataset are currently being utilized by the Mayo Clinic Enterprise Opioid Stewardship Group, which is charged with disseminating opioid prescribing guidelines across all departments at Mayo Clinic. Departments with the highest proportion of prescriptions outside of the state recommended 200 MME are being targeted first for educational efforts to limit overprescribing.

## Major Themes

### Lessons Learned

This project describes innovative use of institutional data sources to answer questions and target quality interventions. We were able to join two institutional datasets to combine information stored in each on prescriptions, services, departments and providers to create heat maps that summarized prescribing practices at our institution and helped to target quality interventions in highlighted areas. We developed an algorithm to create a fuzzy link with dates of service and prescription order date, and standardized prescriptions by converting dosages into MME in order to make reasonable comparisons of prescribing practices. We learned a number of valuable lessons in the process.

A solid understanding of data sources was necessary, both from the front-end system user (i.e., the EOP screen entry that providers use to place in orders), as well as the back-end system users (i.e., how data is stored and pulled from the servers and how information flows from the front end to the back end). In order to do this, it was helpful to involve the database manager from the beginning to assist with data pulls. They were engaged as we worked through data cleaning to remove potential noise in the data that would not suit our purposes such as entry errors, distinguishing between active and canceled orders, and documentation of historical medications obtained through medication reconciliation during an office visit, which may have not been prescribed at our institution.

Clinician and pharmacist engagement was valuable as we worked through cleaning the data and calculating MME conversions. They provided useful feedback during the data pulls, selection of an appropriate opioid conversion tool and providing supplemental conversions to standardize prescriptions. They provided investigation into clinical notes to define and resolve data entry errors or other data nuances for orders with unusually high MME, and validated links with services, departments and providers. They were also instrumental in contextualizing findings and ensuring that findings were consistent with actual clinical practice norms.

Providing ongoing feedback and timelines to guide this work was beneficial for both the stakeholders and the data team. This approach allowed for an understanding of the workflows and complexities of data linkage, as well as troubleshooting and resolution of questions and inconsistencies as they arose. We were able to generate an aggregate view of the departments, and while we are confident in our matching results, a more detailed dive into the data would need to take into consideration the margin of error inherent with the fuzzy link.

### Next Steps

The heat maps will be used to conduct additional deeper dives into data for departments that seem to have higher than expected rates and/or MME of opioid prescribing. We will use elements included in the data to provide additional information including types of prescribing providers, more specific descriptions and locations of services linked to prescriptions, and patient demographics. These data can advance our understanding of the top surgical procedures and medical diagnoses linked with higher doses of opioid prescriptions and enable the practice to assess the degree to which opioids are being appropriately prescribed for these indications.

## Conclusions

Quality improvement efforts are critical for improving the safe and effective delivery of health care services. The current opioids crisis represents an urgent public health crisis compelling health systems and providers to understand current opioid prescribing practices and identify opportunities to improve quality of pain management. Efficient leveraging of existing administrative and clinical data will be required to execute effective practice changes.

Our institutional leadership sought to understand the patterns of opioids prescribing and assess the potential risk of opioid diversion in the community. Our team combined data from accessible institutional data resources to understand the extent of opioid prescribing and determine possible targets for intervention. Heat maps created from these sources indicated departments within each clinical practice setting as potential targets for physician education, and engagement in appropriate use and monitoring of opioid prescriptions.

This case study provides an example of leveraging existing institutional data sources to understand patterns of care and target quality improvement efforts. While this case study was directed at understanding prescribing patterns, the methodology developed and lessons learned in the process used to combine data sources are generalizable can be applied to other health systems and areas of care.
